# Supplementary material for: Detailed molecular and epigenetic characterization of the pig IPEC-J2 and chicken SL-29 cell lines
Source: iScience. 2023 Feb 20;26(3):106252. doi: 10.1016/j.isci.2023.106252 (PMC10018572; doi:10.1016/j.isci.2023.106252)
Supplement: Data S2. Complete homer output for identified motifs in Chicken SL-29, related to Tables 5 and 6 — Homer motif analysis results for histone modifications H3K4me1, H3K4me3, H3K27ac, enhancers, and ATAC-seq of chicken SL-29 cell line. Parameters for possible false positives is as mentioned earlier for S5. [file mmc3.zip › Data_S2/S6/Chicken_SL_29/motif_analyis_enhancer_regions/homerResults/motif6.similar.html]

motif6

## Information for motif6

G
C
T
A
C
T
A
G
T
G
A
C
A
G
T
C
A
T
C
G
A
C
T
G
  
Reverse Opposite:  

A
G
T
C
T
A
G
C
C
T
A
G
A
C
T
G
A
G
T
C
C
G
A
T
  

|  |  |
| --- | --- |
| p-value: | 1e-16 |
| log p-value: | -3.837e+01 |
| Information Content per bp: | 1.765 |
| Number of Target Sequences with motif | 2047.0 |
| Percentage of Target Sequences with motif | 70.22% |
| Number of Background Sequences with motif | 27701.7 |
| Percentage of Background Sequences with motif | 62.80% |
| Average Position of motif in Targets | 142.0 +/- 87.8bp |
| Average Position of motif in Background | 150.3 +/- 123.2bp |
| Strand Bias (log2 ratio + to - strand density) | 0.3 |
| Multiplicity (# of sites on avg that occur together) | 1.07 |
| Motif File: | file (matrix) reverse opposite |

### Similar de novo motifs found

|  |  |  |  |  |  |  |  |
| --- | --- | --- | --- | --- | --- | --- | --- |
| Rank | Match Score | Redundant Motif | P-value | log P-value | % of Targets | % of Background | Motif file |
| 1 | 0.703 | A G T C A G T C A G T C A C T G A G T C A C G T | 1e-13 | -30.862970 | 59.31% | 52.41% | motif file (matrix) |
| 2 | 0.608 | A T G C A G T C A G C T A G T C T A G C T A G C A C T G A T C G G T A C A C G T A C T G G C T A A C T G | 1e-11 | -27.019431 | 0.96% | 0.18% | motif file (matrix) |
| 3 | 0.619 | A T C G T G A C T A G C A T G C T C A G A G T C G A C T A T C G C T G A T C G A | 1e-10 | -23.456290 | 2.50% | 1.06% | motif file (matrix) |
| 4 | 0.601 | T C A G T A G C A G T C A C T G G T A C A G T C A G T C A C T G A C T G G T A C C A T G A C T G T G A C | 1e-9 | -20.962224 | 4.80% | 2.77% | motif file (matrix) |
| 5 | 0.616 | T A G C C A T G T G A C C T A G T G A C C G T A C T A G G T A C C G T A T A C G C A T G A T G C G A T C A T C G A T G C | 1e-8 | -20.331408 | 30.57% | 25.66% | motif file (matrix) |
